# The elusive challenge of priority setting in health and health care

**DOI:** 10.1002/gch2.1008

**Published:** 2015-10-06

**Authors:** Ole F. Norheim

**Affiliations:** ^1^ Department of Global Public Health and Primary Care University of Bergen Bergen Norway; ^2^ Department of Global Health and Population Harvard TH Chan School of Public Health Boston MA USA

## Abstract

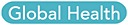

Priority setting in health aims to make peoples' lives go better and that these advantages be distributed fairly. The scope is broad. Health is a key element of well‐being and therefore not only important in itself but also necessary for pursing a good life. Population health is improving all over the world. Global life expectancy at birth has increased with almost three months every year in the last decades. Healthy life expectancy is also increasing rapidly. Many of us live longer and better lives.

But these improvements are distributed unequally; there are still large inequalities in healthy life expectancy between the poorest and richest countries and between the poorest and richest groups within countries. Among the global poor, the bottom billion, lack of universal access to essential services is perhaps the greatest unfairness. In middle‐income countries, expensive new technologies put pressure on the system. Disease group inequality also exists within countries. Even in high‐income countries, lifetime health for patients with schizophrenia or multiple sclerosis is much lower than for most patients with coronary heart disease or testicular cancer. Factors such as genetic endowments, social circumstances, unequal advances in technology, and unequal access to services may explain such inequalities. Fair distribution aims to reduce them.

And yet, priority setting is an elusive theme in national and global health policy. First, even if better priority setting is in the interest of all, it is not obvious that it is in the interest of every decision‐maker. Priority setting in health care can be defined as the ranking of health services and the ranking of recipients of these services. The ranking of services or patients can be systematic and evidence based, or arbitrary and ad hoc, and is typically a mixed result of planned policies, financing mechanisms, historical budgets, legal regulations, the interests of health professionals, and the influence of patient organizations and public opinion. Given resource scarcity, better priority setting implies withholding interventions to some patients or not investing in the lowest ranked health services, even if they are marginally beneficial – on the grounds that resources could be better and more fairly spent elsewhere. Making such decision will always be controversial and unpopular. Second, there is often ethical and public disagreement about the standards of evaluation. Reasonable people disagree about what is fair. This is for some another reason to avoid the hard choices. They seek technical solutions to what is at heart an issue of distributive justice.

Despite this, the need for better, more fair, and legitimate priority setting will not go away. Decision‐makers – and all of us as citizens – need the contributions from academic research. We need to better understand clinical and political decision‐making; how to strengthen national and global intuitions for better priority setting; to know whether or how incentive mechanisms should be changed; and to identify the proper roles for legal regulation, institutional obligations, and individual health rights. We need evidence about which policy instruments and health interventions work; how large their benefits and costs are; and their impact on health inequalities. Decisions‐makers, academics, and the public also need better understanding of the underlying ethical questions: How to evaluate improvements in population health; whether we can agree on a set of necessary and a set of unacceptable criteria for priority setting; how to reconcile the tensions between individual claims and population health, and whether substantive distributive principles can be integrated with frameworks for fair and legitimate process.

The academic field of priority setting in health and health care is now fairly well established. Seminal papers and books started to appear 30–40 years ago. Even Ken Arrow's 1963 paper on market failures in health insurance is still highly relevant (Arrow, 1963). Other early contributions from decision theory and health economics laid out the foundations for cost‐effectiveness analysis, gradually refining its methods, scope, and relevance (Weinstein and Stason, 1977; Williams, 1985; Eddy, 1990; Culyer and Wagstaff, 1993; Drummond et al., 1997; Nord, 1999; Dolan and Olsen, 2002). In ethics, inspired by John Rawls' theory of distributive justice, early contributions clarified the arguments for a moral right to universal health care and identified key principles, criteria, and conditions for legitimate priority setting (Daniels, 1985; Kamm, 1987; Broome, 1988; Anand, 2002; Sen, 2002; Brock and Wikler, 2006). Seminal studies in health policy clarified the role of institutions and the complex patterns of priority setting (Klein and Redmayne, 1994; Ham and Coulter, 2003). A more recent field is the study of law and the impact of courts on the distribution of health and health care (Yamin and Gloppen, 2011).

Some countries early on saw the need for national guidelines on priority setting. The first came in Norway in 1987 followed by the Netherlands, Sweden, and New Zealand. The National Institute for Health and Care Excellence in UK has been the most visible institution, also recognized internationally, for pioneering systematic health technology assessment, cost‐effectiveness analysis, and clinical guidelines development while also seeking input from the public through their citizen's panel. Another institution that made a long‐lasting early contribution to the field was the World Bank. In 1993, the first edition of Disease Control Priorities in Developing Countries and the report Investing in Health were published (Jamison et al., 1993; World Bank, 1993). The third edition of Disease Control Priorities is now well underway. Later, the World Health Organization established the WHO‐CHOICE database. Recently, the National Institute for Health and Care Excellence (NICE) International launched the international Decision Support Initiative to support low‐income and middle‐income governments in making resource allocation decisions for health care. Finally, there is the International Society on Priority Setting in Health Care, established in 1996, that organizes biannual conferences.

Now, a new generation of academics should explore the challenges of priority setting. Despite all the positive developments in health and the institutionalization of priority setting, the elusive challenge of priority setting will not disappear. We should persuade decision‐makers to embrace priority setting, not evade it. Innovations and new approaches are welcome. This journal invites all types of manuscripts on priority setting in health and health care, especially those that are interdisciplinary, critical, constructive, and global in reach. Our aim should be, as it was once said about the Indian economist and philosopher Amartya Sen, to bring “arguments for a better world” (Basu and Kanbur, 2008).
